# Mendelian randomization studies on coronary artery disease: a systematic review and meta-analysis

**DOI:** 10.1186/s13643-023-02442-8

**Published:** 2024-01-16

**Authors:** Sarah Silva, Segun Fatumo, Dorothea Nitsch

**Affiliations:** 1 Department of Non-Communicable Disease Epidemiology, London School of Hygiene and Tropical Medicine, London, UK; 2 The African Computational Genomics (TACG) Research Group, MRC/UVRI, and LSHTM, Entebbe, Uganda

**Keywords:** Coronary artery disease, Coronary heart disease, Ancestry, Mendelian randomization, Systematic review

## Abstract

**Background:**

Coronary artery disease (CAD) remains one of the leading causes of mortality worldwide. We aimed to summarize what is currently known with regard to causal modifiable risk factors associated with CAD in populations of diverse ancestries through conducting a systematic review and meta-analysis of Mendelian randomization (MR) studies on CAD.

**Methods:**

The databases Embase, Medline, Cochrane Library and Web of Science were searched on the 19th and 20th of December 2022 for MR studies with CAD as a primary outcome; keywords of the search strategy included “coronary artery disease” and “mendelian randomization”. Studies were included if they were published in the English language, included only human participants, employed Mendelian randomization as the primary methodology and studied CAD as the outcome of interest. The exclusion criteria resulted in the removal of studies that did not align with the predefined inclusion criteria, as well as studies which were systematic reviews themselves, and used the same exposure and outcome source as another study.

An ancestry-specific meta-analysis was subsequently conducted on studies which investigated either body mass index, lipid traits, blood pressure or type 2 diabetes as an exposure variable. Assessment of publication bias and sensitivity analyses was conducted for risk of bias assessment in the included studies.

**Results:**

A total of 1781 studies were identified through the database searches after de-duplication was performed, with 47 studies included in the quantitative synthesis after eligibility screening. Approximately 80% of all included study participants for MR studies on CAD were of European descent irrespective of the exposure of interest, while no study included individuals of African ancestry. We found no evidence of differences in terms of direction of causation between ancestry groups; however, the strength of the respective relationships between each exposure and CAD were different, with this finding most evident when blood pressure was the exposure of interest.

**Conclusions:**

Findings from this review suggest that patterns regarding the causational relationship between modifiable risk factors and CAD do not differ in terms of direction when compared across diverse ancestry populations. Differences in the observed strengths of the respective relationships however are indicative of the value of increasing representation in non-European populations, as novel genetic pathways or functional SNPs relating to CAD may be uncovered through a more global analysis.

**Systematic review registration:**

The protocol for this systematic review was registered to the International Prospective Register of Systematic Reviews (PROSPERO) and is publicly available online (CRD42021272726).

**Supplementary Information:**

The online version contains supplementary material available at 10.1186/s13643-023-02442-8.

## Introduction

Cardiovascular diseases are the leading cause of mortality worldwide, with a global burden of 17.9 million deaths in 2021 [1]. Despite ongoing efforts towards its prevention and treatment, coronary artery disease (CAD) remains the most prevalent and fatal of the cardiovascular diseases [2–4]. Gaining insight into the complex aetiology of CAD, involving a dynamic relationship between genetic and environmental factors, is vital in developing early intervention strategies for its prevention [3, 5, 6]. Although it is well established that the majority of the CAD burden is associated with modifiable risk factors, understanding of the causal relationships between risk factors and CAD, beyond associations identified in epidemiological studies, needs to be improved in order to refine and develop a more global understanding of the pathophysiology of the disease [4, 7, 8].

Widely considered the gold standard for studying the causal relationships between interventions and outcomes, randomized controlled trials (RCTs) reduce bias observed in many other study designs [9, 10]. With this however, the difficulty of disentangling causation from correlation in observational studies, especially in the presence of residual confounding and reverse causality, introduces bias and consequently limits causal inferences of RCTs [11–13]_._ The Mendelian randomization (MR) epidemiological methodology aids in overcoming these limitations through exploiting naturally occurring genetic variants as proxies for modifiable risk factors, in order to determine the causal association of selected exposures on outcomes of interest [14–16]. The MR study design is less susceptible to confounding and reverse causality, as it is based on one’s random allocation of genetic variants at birth, which precedes the phenotype of interest, cannot be changed by the phenotype and is not influenced by external factors [3, 17–19]. These genetic variants, referred to as instrumental variables (IVs), are used to assess the association of a genetically predicted exposure with an outcome of interest, which in this case is CAD.

In order for an MR analysis to be valid, it necessitates that three IV assumptions must be met. Firstly, that the genetic variants used as proxies for the instrumental variable are associated with the exposure of interest, secondly, that the genetic variants are not associated with any known or unknown confounders, and lastly, that the genetic variants are not independently associated with the outcome of interest other than through the selected exposure [12, 20, 21].

Coinciding with the proliferation of data availability from genome-wide association studies (GWAS), the employment of MR as a method to assess causality has substantially increased. With this however, the majority of available GWAS data, and therefore MR evidence, has been derived from populations of European ancestry. This gives rise to questions regarding the generalizability of findings to non-European populations and the translation of findings on a global scale [3, 5, 22].

Understanding the scope of the current research landscape, with a focus on populations of diverse ancestries, will allow for advances in our epidemiological insights of CAD and highlight causal relationships and risk factors which require further research. Recognizing ancestry-specific patterns and variations of disease causation will expectantly help shape policy development and precision medicine, through tailoring treatment and prevention strategies to populations where respective risk factors have a higher causal relationship with CAD than others. This will additionally contribute towards achieving more equitable healthcare amongst diverse populations and allow for a more global understanding of CAD.

In this paper, we systematically reviewed the literature for studies investigating the causal relationship between genetically predicted modifiable risk factors and CAD using MR methodology. We aimed to summarize and describe what is known with regard to causal modifiable risk factors associated with CAD in diverse populations. Additionally, we aimed to identify patterns of causal effects that may exist between populations of different ancestries in order to establish how much we currently understand about the genetic architecture of CAD on a global scale.

## Methods

This systematic review was conducted in accordance with the Preferred Items for Systematic Reviews and Meta-Analysis (PRISMA) guidelines [22]. The corresponding protocol for the systematic review was registered to the International Prospective Register of Systematic Reviews (PROSPERO) and is publicly available online (CRD42021272726).

### Search strategy

Published studies in Embase, Medline, Cochrane Library and Web of Science were searched for and extracted on the 19th and 20th of December 2022. A pre-defined search strategy was employed, using a combination of the terms “mendelian randomization”, “genetic instrument” and “coronary artery disease”, as well as their corresponding synonyms and keywords. Using database search filters, we restricted the results to include only human participants and studies published in the English language. We chose to not implement any date restrictions in the search as Mendelian randomization is a relatively new methodology and therefore ran respective searches from the date of inception of each database. The complete search strategies employed for each respective database is provided in Supplementary Material 1. Additionally, the reference lists of the included studies were screened in order to identify any additional references that were not picked up in the search.

### Eligibility criteria

Once identified, studies were included if they (i) were published in the English language, (ii) included only human participants, (iii) employed Mendelian randomization as the primary methodology and (iv) had CAD as a primary outcome of interest. We excluded studies which (i) were systematic reviews themselves, (ii) included non-human subjects in the study, (iii) did not employ Mendelian randomization as the primary research methodology, (iv) did not investigate CAD as an outcome of interest, (v) used the same exposure and outcome data sources as another study for the analysis and (vi) were duplicated across databases.

### Data extraction

An extraction template was developed based on the PRISMA guidelines (Supplementary Material 2). The following information was collected from each study: first author’s name, year published, title, ancestry of participants, type of MR study, exposure data source(s), exposure variable(s), exposure sample size(s), number of single-nucleotide polymorphisms (SNPs) used, number of proxy SNPs used, independent SNPs tested, outcome data source(s), outcome variable(s), outcome sample size(s), MR analysis results, types of sensitivity test(s) conducted, sensitivity test results, F-statistic, heterogeneity, power calculations, confounders tested.

### Quality assessment

After identified studies were de-deduplicated, a random sample of 100 studies was independently evaluated by the three authors of this paper for inclusion or exclusion decisions, following the pre-determined eligibility criteria. Any differences in decisions were resolved in a discussion between all three authors. This was performed in order to calibrate the inclusion and exclusion judgements from all authors and serve as a reference for the remaining screening steps which were performed by a single author (obtained kappa coefficient = 0.91). For quality assessment, information was extracted based on a template developed from the Strengthening the Reporting of Observational Studies in Epidemiology using Mendelian Randomization (STROBE-MR) guidelines [23]. Publication bias and heterogeneity were additionally analysed using the R programming language for each study included in the meta-analysis ([24]; Supplementary Material 5–6).

### Meta-analysis

Primary outcomes were recorded as odds ratios (OR) with 95% confidence intervals, with data pooled into a meta-analysis when at least two or more studies investigated the same exposure variable on CAD in respective ancestry groups. The ancestry-specific meta-analyses for this systematic review were performed using the “meta” package in the R programme version 4.2.3, where all related figures were created as well [24, 25]. All reported estimates are expressed per standard deviation increase in genetic liability to CAD, with comparisons between ancestry groups shown in the same plot.

## Results

### Identified studies in the systematic review

**Fig. 1:**
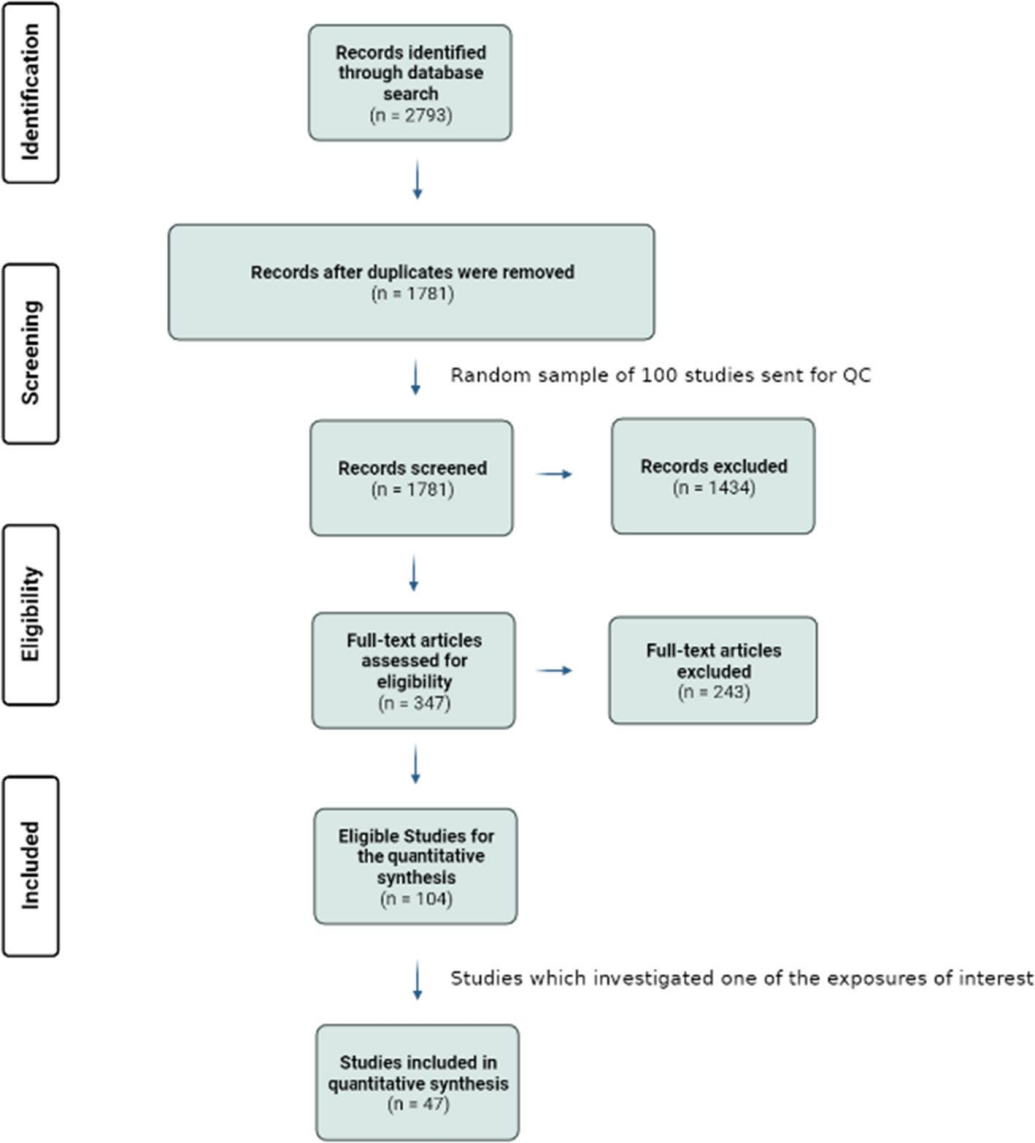
Preferred reporting items for systematic reviews and meta-analyses flowchart for the systematic review of Mendelian randomization studies of coronary artery disease

The search strategy identified a total of 2793 relevant studies, from which 1012 duplicates were removed. Title and abstract screening excluded an additional 1434 studies, with the main reasons for exclusion being different MR outcome variable besides CAD, use of a study methodology besides MR and abstract-only papers (Fig. 1). Full-text screening resulted in the exclusion of 243 studies, leaving 104 eligible studies to be included in the quantitative synthesis. Reasons for exclusion after full-text screening included: outcomes were measured as combined cardiovascular disease outcomes instead of CAD outcomes alone, the definition of CAD employed in the study was vague compared to the case definition used in other studies, and that the assessment of casual associations was mentioned in either the title or abstract; however, the MR methodology was not employed.

The main exposures of interest for this systematic review and meta-analysis were body mass index (BMI), blood pressure, lipid traits and type 2 diabetes mellitus (T2DM). Therefore, after identifying only studies investigating one of these risk factors as an exposure, 45 studies were left to be included in this systematic review [11, 12, 15–17, 19, 20, 26–64].

### Exposures of interest with CAD as an outcome

**Fig. 2:**
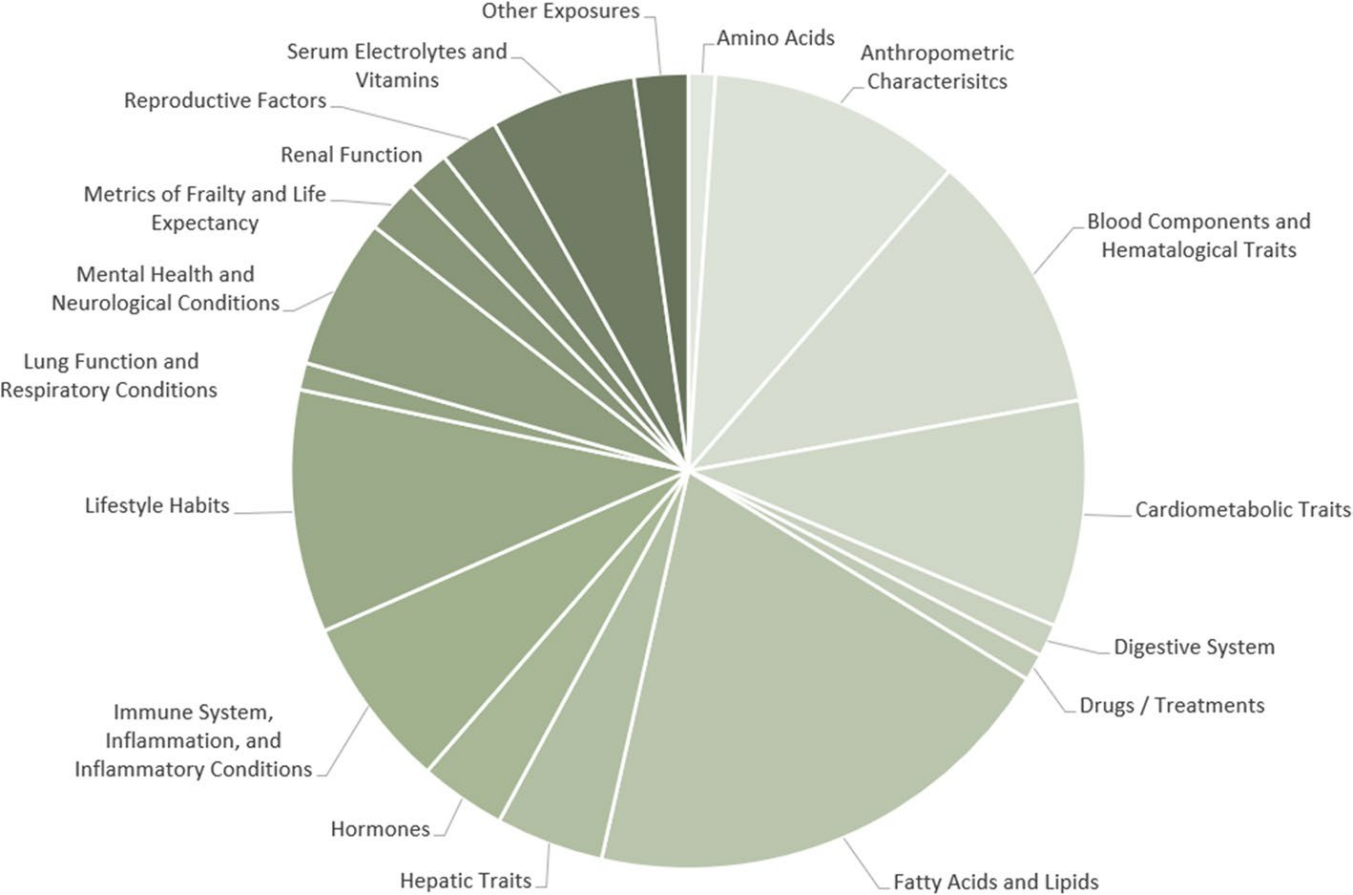
All exposures studied in Mendelian randomization studies with coronary artery disease as an outcome

Before narrowing down full-text articles to be included in the quantitative synthesis, all of the different exposures investigated in each eligible MR study with CAD as an outcome were noted. Exposures were then grouped into similar categories in order to better visualize the current MR research landscape with CAD as an outcome (Fig. 2; Supplementary Material 3).

Almost 20% of all MR studies conducted with CAD as an outcome in the current research landscape investigated fatty acids or lipid traits as an exposure. Eleven percent of studies investigated blood components and haematological traits as an exposure, while nearly 10% studied anthropometric characteristics, cardiometabolic traits and lifestyle habits respectively. As mentioned previously, due to the large number of studies available, the exposures selected to be studied in the context of population ancestries for this systematic review were BMI, blood pressure, lipid traits and T2DM.

### Ancestries of participants for studies with BMI, lipid traits, blood pressure or type 2 diabetes mellitus as exposure variables

**Fig. 3:**
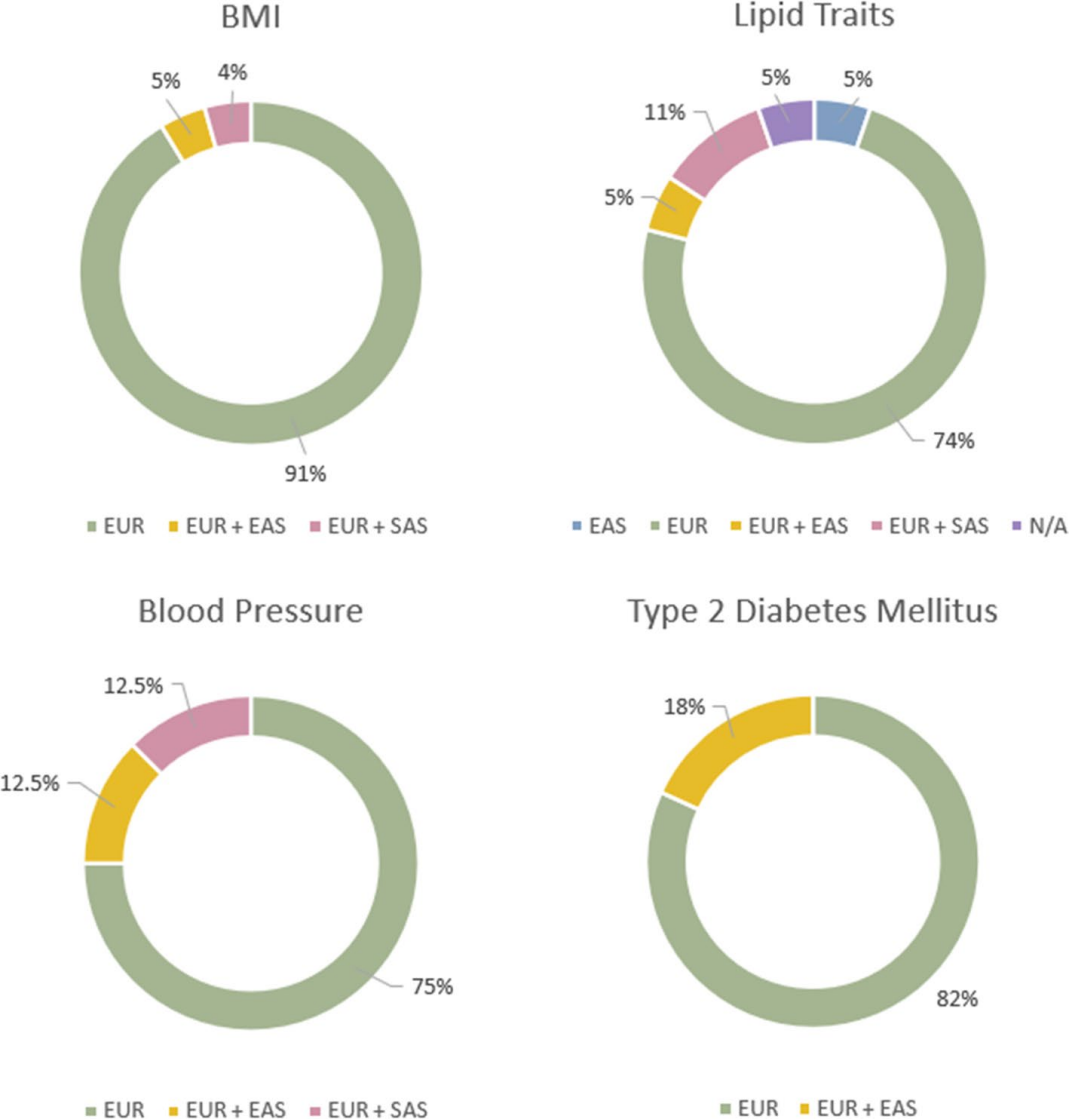
Distribution of ancestry populations included in Mendelian randomization studies of coronary artery disease, with BMI, blood pressure, lipid traits and type 2 diabetes mellitus used as exposures respectively

The ancestries of the populations included in each of the four exposure categories of interest were compared against each other (Fig. 3). BMI was the most frequently studied exposure, followed by lipid traits, T2DM and blood pressure. Six of the included studies investigated more than one of the exposures of interest in the same MR study and approximately 80% of the participants included in each exposure category were of European ancestry, with East Asian being the only other ancestry group included for each exposure variable. Only one study included a non-European ancestry group which was investigated independently from a European cohort, while no study included individuals of African ancestry.

### Quality assessment of included MR studies

The quality assessment information and results from studies included in this review are shown in Supplementary Material 4. The majority of studies employed a two-sample MR approach, employing inverse-variance weighted analysis for the instrumental variable analysis. Sixty-seven percent of the studies further performed a sensitivity analysis and an additional 15% of studies employed methods besides formal statistical techniques as a form of sensitivity analysis.

Less than half of the studies (37%) clearly verified all three key IV assumptions. A limited number of studies were unclear with how each of the IV assumptions was validated; 46% of the studies addressed possible confounding, while 83% reported an F-statistic and 91% discussed potential pleiotropy. All studies addressed their respective study limitations and interpreted the meaning of their MR results (100%); however, only 59% specifically discussed the generalizability of MR results.

The reporting of statistical power was frequently overlooked in the included studies, with studies either not including power calculations at all (59%) or being unclear with power results (4%). However, with clear participant summary statistics (93%) and primary MR results (100%), inferences regarding the statistical relevance of the data were still made.

### Meta-analysis

The direction of causality from the MR results was generally observed to be similar across all ancestries. Of all exposures, only HDL-C is seen to be protective against CAD, with an increase in HDL-C associated with a lower risk of CAD; although limited again by levels uncertainty, this relationship is observed across all ancestry populations. The study investigating the causal effect of systolic blood pressure on CAD in South Asians was the only study which yielded results indicative of a different genetically predicted causal relationship compared to other ancestries; however, the size of the confidence intervals limits the ability to make a conclusion on the direction of the relationship. Additionally, the most variability in odds ratios between ancestry groups can be seen when either diastolic and systolic blood pressure were employed as exposure variables, where the 95% confidence intervals were largest for these exposures, possibly due to the variability of blood pressure measurements and the sample sizes of the included studies.

**Fig. 4:**
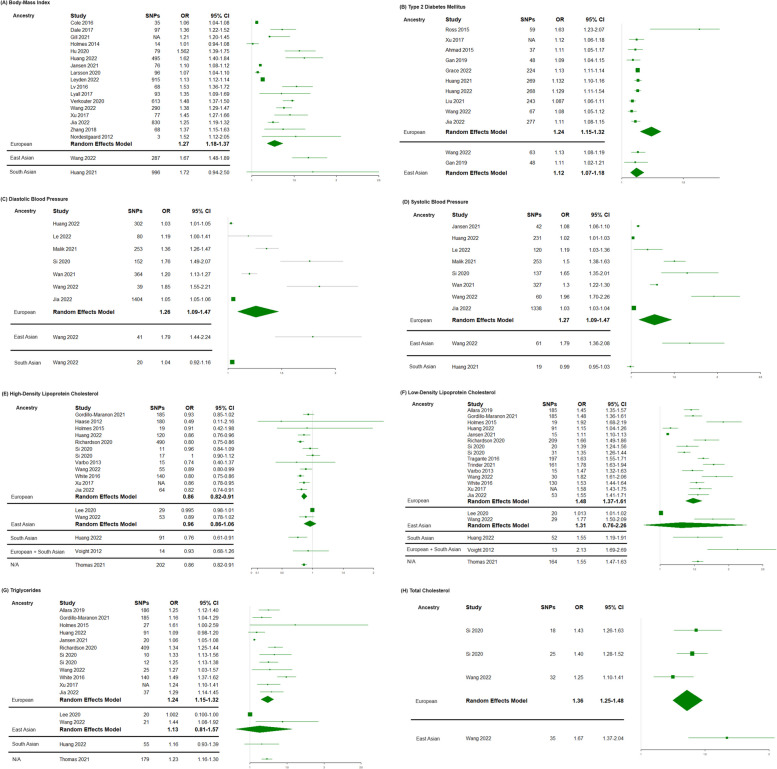
Meta-analysed forest plots for BMI, blood pressure, lipid traits and type 2 diabetes mellitus with distinctions between ancestry groups. **A** BMI. **B** Type 2 diabetes mellitus. **C** Diastolic blood pressure. **D** Systolic blood pressure. **E** High-density lipoprotein-cholesterol. **F** Low-density lipoprotein-cholesterol. **G** Triglycerides. **H** Total cholesterol

For diastolic blood pressure in each of the European, East Asian and South Asian ancestry groups, the summarized OR and 95% CI were as follows: 1.26 (1.09–1.47), 1.79 (1.44–2.24) and 1.04 (0.92–1.16) respectively. For systolic blood pressure, studies conducted in individuals of European ancestry had a meta-analysed OR of 1.27 (95% CI; 1.09–1.47), East Asians had an OR of 1.68 (95% CI; 1.36–2.08) and South Asians had an OR of 0.99 (95% CI; 0.95–1.03). Despite these large confidence intervals, there is no overlap between East Asian and South Asians, meaning that the genetic liability to blood pressure increases the risk of CAD significantly more in East Asians than South Asians (Fig. 4).

## Discussion

To the best of our knowledge, this is the first systematic review focusing on understanding ancestry-specific differences between the causal effects of exposures on CAD through MR studies [30, 31, 43, 61]. Employing a comprehensive and reproducible search strategy, we searched and mapped the current literature to get an idea of what types of research has been done with regard to MR studies with CAD as an outcome, and further focused on evaluating the associations of four different exposures on CAD in the context of ancestry. BMI, blood pressure, lipid traits and T2DM were selected as exposures of interest as they are all traditionally well-known risk factors for CAD. This allowed for comparisons to be made across ancestry populations, while ensuring relatively consistent MR results within ancestry groups where research has previously been conducted.

### Systematic review

Although no evidence of differences was observed in terms of the direction of the effects between ancestries in this study, the magnitude of the genetically governed associations between each respective exposure and outcome were different when compared between each ancestry population; this was most evident in MR studies investigating the association of systolic and diastolic blood pressure on CAD. The observed differences in the magnitude of effects between ancestry groups highlighted the possibility that different ancestries have different patterns of genetically determined causal effects. It is of note however that the variability observed in the OR values for studies conducted in individuals of South Asian ancestry, due to levels of uncertainty, limits the confidence of a genetically predicted association of blood pressure with the risk of CAD, as the study was too small and potentially included incorrect instrumental variables. On the other hand, the high OR values observed in East Asians were consistent with previous results suggesting that increases in blood pressure affect individuals of East Asian ancestry greater than others [65, 66].

This observed genetic variation in the risk factors for CAD between populations further highlights the benefits of increasing diversity in order to expand the current understanding of human genomic variation and its contribution towards disease pathophysiology. If differences do exist in the genetic architecture of CAD worldwide, the disproportionate disease burden observed in non-European ancestry groups can begin to be addressed through developing precision-based prediction, prevention and treatment opportunities for CAD based on population-specific evidence.

### Importance of including diverse populations in research

Recognizing differences exist in the risk factor burden, incidence and prevalence of CAD between ancestries has resulted in the widespread acknowledgement that increasing diversity amongst study participants is essential in order to continue advancing our understanding of the genetic architecture and pathophysiology of CAD from a global perspective [7, 29, 67].

Although traditional epidemiological studies have established the fact that CAD prevalence and death rates vary between ancestry groups, with the highest observed amongst individuals of South Asian ancestry, insufficient research in such populations leaves a lot of uncertainty regarding disease pathophysiology [43, 68–70]. The current research landscape is further hindered by that fact that it predominantly involves cohorts of non-European populations who are immigrants living in Western countries, instead of collecting and using data from regions around the world. This limits the generalizability of findings as researchers are unsure as to what extent findings apply worldwide. From research which has been conducted comparing individuals from different global regions to date, small but population-relevant differences between risk factors and CVD outcomes have been identified [66, 71–73]. This suggests that significant differences may be identified in CAD outcomes if diverse studies with increased representation are carried out. The complex interactions between genes and the environment greatly contribute towards the observed varied genetic association between exposures and an outcome.

Limited representation hinders the ability to confidently compare results with sufficient power. Although differing patterns of genetic association can be observed between populations of different ancestries from this systematic review, increasing the representation of non-European ancestries in the research landscape would allow for the observation of the association of instruments with CAD in different environmental contexts, and subsequently aid in developing a global understanding of the complex relationship between modifiable risk factors and the genetic basis of the disease.

### Limitations

Despite this being the first large-scale ancestry-specific systematic review carried out on MR studies with CAD as an outcome, a number of limitations should be acknowledged. The search strategy and eligibility criteria of this systematic review may have resulted in us overlooking some potentially relevant studies which employed MR as a supplementary analysis instead of the primary focus. This type of study would have expectantly not addressed the MR instrumental variable assumptions as much as if it was a primary research methodology; however, it may have contributed towards more representation and findings nonetheless.

Additionally, we only included full-text published studies in this review. By doing this, we restricted findings which were shared through other means of publication and consequently contributed towards the potential omission of relevant studies. The exposures chosen for the investigation represent those that are most understood and are not representative of the entire research landscape. Despite identifying many more studies employing the MR design to investigate CAD as an outcome, we chose to focus on only four exposures for this paper. This methodology was employed in order to effectively compare patterns of genetic variation across ancestry groups, although a comparison on a much larger scale would have also been possible.

Finally, while these findings provide insights into the causal effects of the exposure traits, the true magnitude of the effects cannot be accurately assessed. This consequently limits the utility of findings, specifically with regard to assessing the impact of an intervention developed based on MR results on the clinical outcome. Estimates derived from MR studies are most beneficial when used in the context of findings from other epidemiological studies and therefore should not be interpreted superficially or independently.

## Conclusion

This systematic review suggests that patterns for the causal risk factors associated with CAD are generally the same across ancestries, with increased levels of BMI, T2DM, blood pressure and lipid traits all being causational towards increased CAD risk, except for HDL-C which has a protective relationship. We highlighted the disproportionate representation of diverse ancestry populations in the current research landscape, most notable observed in populations of African ancestry, and believe that increasing representation of non-European population groups in future research will allow for the identification of novel causal pathways relating to CAD. We expect that as MR methodologies continue advancing in congruency with the increasing interest in investigating genetic diversity in different populations, a more global understanding of the pathophysiology of CAD can be developed in order to compliment findings from traditional observational studies.

### Supplementary Information

**Additional file 1.**

## Data Availability

All data generated or analysed during this study are included in publicly available databases, within this published article and within its supplementary information files.

## Abbreviations

CAD: Coronary artery disease
MR: Mendelian randomization
RCTs: Randomized controlled trials
IVs: Instrumental variables
GWAS: Genome-wide association studies
PRISMA: Preferred Items for Systematic Reviews and Meta-Analysis
PROSPERO: International Prospective Register of Systematic Reviews
STROBE-MR: Strengthening the Reporting of Observational Studies in Epidemiology using Mendelian Randomization
OR: Odds ratios
BMI: Body mass index
T2DM: Type 2 diabetes mellitus
HDL-C: High-density lipoprotein cholesterol
